# YOLO-DHGC: Small Object Detection Using Two-Stream Structure with Dense Connections

**DOI:** 10.3390/s24216902

**Published:** 2024-10-28

**Authors:** Lihua Chen, Lumei Su, Weihao Chen, Yuhan Chen, Haojie Chen, Tianyou Li

**Affiliations:** 1 School of Electrical Engineering and Automation, Xiamen University of Technology, Xiamen 361024, China; 2322131006@stu.xmut.edu.cn (L.C.); chenweihao0718@163.com (W.C.); yuhanchen_work@163.com (Y.C.); 2322131004@stu.xmut.edu.cn (H.C.); ltyxm@163.net (T.L.); 2 Xiamen Key Laboratory of Frontier Electric Power Equipment and Intelligent Control, Xiamen 361024, China

**Keywords:** small object detection, two-stream structure, dense connection

## Abstract

Small object detection, which is frequently applied in defect detection, medical imaging, and security surveillance, often suffers from low accuracy due to limited feature information and blurred details. This paper proposes a small object detection method named YOLO-DHGC, which employs a two-stream structure with dense connections. Firstly, a novel backbone network, DenseHRNet, is introduced. It innovatively combines a dense connection mechanism with high-resolution feature map branches, effectively enhancing feature reuse and cross-layer fusion, thereby obtaining high-level semantic information from the image. Secondly, a two-stream structure based on an edge-gated branch is designed. It uses higher-level information from the regular detection stream to eliminate irrelevant interference remaining in the early processing stages of the edge-gated stream, allowing it to focus on processing information related to shape boundaries and accurately capture the morphological features of small objects. To assess the effectiveness of the proposed YOLO-DHGC method, we conducted experiments on several public datasets and a self-constructed dataset. Exceptionally, a defect detection accuracy of 96.3% was achieved on the Market-PCB public dataset, demonstrating the effectiveness of our method in detecting small object defects for industrial applications.

## 1. Introduction

Small object detection, which is a key technology in fields such as industrial defect detection, remote sensing image monitoring, and drone inspection, is a major research focus in computer vision [1,2,3]. Early small object detection primarily relied on traditional machine vision techniques, which included rule presetting, image processing, and feature matching, to identify and locate objects [4]. However, small objects often have diverse shapes, and traditional machine learning methods that rely on handcrafted features often struggle to achieve optimal detection performance.

With the rapid advancement of deep learning technology, many researchers are exploring small object detection algorithms [5,6,7] based on deep learning. Compared to traditional machine learning methods, deep learning-based small object detection methods automatically extract and recognize features by convolutional neural networks without manual feature design. Consequently, the detection accuracy of deep learning-based methods is higher. Based on these advantages, researchers have improved small object detection algorithms by focusing on data augmentation [8,9,10], multi-scale feature fusion [11,12,13], incorporating contextual information [12,14,15,16], designing anchor mechanisms [17,18,19], and using generative adversarial networks [20,21,22]. The accuracy and robustness of small object detection are enhanced by these efforts.

Some research [11,12,13] utilizes integrated future techniques such as pyramid structure or jump connection to integrate different levels of feature maps. These techniques enhance the model’s sensitivity to small object details by extracting multi-scale features from small to large. Some research [12,14,15,16] introduces contextual information by expanding the receptive field and using attention mechanisms. These methods enable the model to reference environmental information around small objects, which helps distinguish subtle differences in similar backgrounds and improves the accuracy of small object detection. Other research includes using data augmentation to enrich sample diversity [8,9,10], optimizing anchor box parameters to improve recall [17,18,19], and generating high simulation samples with the help of GANs [20,21,22] to enhance small object detection accuracy.

Accuracy of small object detection is improved by the methods mentioned above, but certain challenges remain. First, it is difficult to extract effective discriminative features for small objects. During feature extraction, the spatial dimensions of the image are reduced after convolution and pooling operations. As a result, the feature representation of small objects in the feature map becomes sparser and may even be completely lost. The lack of discriminative features directly affects recognition accuracy [23]. Second, it is difficult to accurately locate small objects in images with complex backgrounds. In conventional detection processes, object detection models integrate various information such as shape, color, and texture. This integration makes the ability of the target detection model to learn different features difficult. In small object detection tasks, it is crucial to accurately capture the boundary information of small objects. However, the receptive field information in the feature map becomes averaged and mixed with background noise after convolution and pooling. Therefore, localization accuracy is reduced because it is difficult to extract the boundary information of small objects

To address the above challenges, this paper proposes a small object detection algorithm called YOLO-DHGC. This algorithm combines a two-stream structure based on an edge-gated branch with the high-resolution backbone network DenseHRNet. The boundaries and shape features as well as detailed appearance information of small objects are captured. Then, the accuracy of precise identification and localization in complex backgrounds is improved.

In summary, the main contributions of the work in this paper are as follows:

(1) We designed a two-stream structure based on an edge-gated branch, which includes a regular detection stream and an edge-gated stream. The regular detection stream uses the DenseHRNet backbone to capture high-level semantic information from the image. An edge-gated stream is especially proposed to extract object contour information of small objects in images with complex backgrounds. The localization accuracy of small objects is improved by emphasizing boundary information and reducing background interference;
(2) We designed a feature extraction backbone network called DenseHRNet. By incorporating a dense connection mechanism, the network extracts and transmits feature information across multiple layers in the main pathway of high-resolution feature maps. This proposed mechanism compensates the loss of the detailed information of objects, which occurs due to reduced image resolution after downsampling. As the backbone of the regular detection stream, DenseHRNet uses a dense connection mechanism to transmit feature information across multiple layers, thereby improving the accuracy of small object recognition;
(3) To further verify the performance of our method, we constructed a dataset of backlight panel images with surface micro-defects captured in a real industrial production line. This dataset was used to test and validate the generalization performance of the YOLO-DHGC algorithm.

The structure of this paper is as follows:

Section 2, Related Work, reviews existing research related to small object detection and discusses the importance of dense connection mechanisms in deep learning model design, as well as the application of two-stream structures in this field.

Section 3, Methods, provides a detailed description of the YOLO-DHGC algorithm’s overall framework and its main components, including the feature extraction module, feature fusion module, and the design details of the detection head.

Section 4, Experiments, discusses the experimental setup, the datasets used, and the performance of our method on multiple benchmarks.

Section 5, Conclusion, summarizes the main findings of this study and explores future research directions.

## 2. Related Work

### 2.1. Small Object Detection

Small object detection methods based on deep learning can be categorized into five main types: (a) Multi-scale feature fusion: This approach integrates feature maps from different levels, combining shallow details with deep semantic information. For example, MSAFFNet by Tong et al. [24] uses dual attention modules (DAMs) and multi-scale feature fusion to enhance detection performance. Tang et al. [25] proposed HIC-YOLOv5, which improves small object detection performance by adding a prediction head specifically designed for small targets, using involution blocks, and incorporating the CBAM attention mechanism at the end of the backbone network. (b) Introducing contextual information: By capturing relationships between objects, this method compensates the lack of pixel information in small objects. AGPCNet by Zhang et al. [26] improves infrared small object detection performance through multi-scale context information fusion. (c) Small object data augmentation: This improves detection performance by increasing the quantity and diversity of small objects in the dataset. The TBi-YOLOv5 model by Huang et al. [9] employs a random augmentation strategy to significantly enhance detection capabilities. (d) Anchor box design: The generation method of preset boxes is optimized to match the size and shape of small objects. Dai et al. [27] proposed a full-scale pseudo-box label assignment scheme combined with a cascade refinement framework to improve detection accuracy. (e) Application of generative adversarial networks (GANs): By generating high-resolution samples, this method enhances both the dataset and detection performance. Bosquet et al. [20] proposed an integrated data augmentation pipeline using DS-GAN to create high-quality synthetic data for small objects, significantly enhancing detection performance.

These methods collectively advance the development of small object detection technology, providing effective solutions for practical applications.

### 2.2. Two-Stream Structure in Spatial and Temporal Feature Extraction

The two-stream architecture, which is often used to process spatial and temporal information or data from different modalities separately, has gained widespread attention as an effective feature extraction mechanism. By separately processing spatial and temporal information or data from different modalities, this architecture enables the system to understand input data more comprehensively, thereby enhancing detection accuracy and robustness.

Simonyan et al. [28] proposed a two-stream ConvNet architecture consisting of spatial and temporal networks, which improves action recognition performance in videos by separately processing appearance and motion information. Li et al. [29] introduced a novel two-stream Convolutional Human Activity Transformer (THAT) model. It captures time-over-channel and channel-on-time features and uses multi-scale convolution to enhance transformers, improving performance in WiFi-based HAR tasks. Cheng et al. [30] proposed a two-stream IF algorithm based on deep features, combining spectral and spatial information. They introduced neural networks to enhance the IF model, fusing spatial detection maps from morphological attribute filtering and Gaussian filtering with spectral results, thereby improving hyperspectral anomaly detection performance. Yi et al. [31] proposed a two-stream gated progressive optimization network called GPONet for salient object detection. By designing a multi-level feature fusion module with a gating mechanism (GFN) and introducing detail-aware loss to guide the model to focus on edge information, the detection accuracy is improved.

The two-stream architecture is typically used to address the challenge of integrating spatial and temporal information or data from different modalities, enhancing the system’s understanding and performance. We innovatively apply this architecture to small object detection, incorporating an edge operator to capture gradient information for improved boundary detection.

### 2.3. Dense Connections for Feature Reuse

In recent years, dense connections have become an important concept in deep learning model design to fully utilize feature reuse and information flow within networks. By directly connecting each layer to all subsequent layers, dense connections promote gradient transmission, reduce the vanishing gradient problem, and enhance feature propagation and reuse, thereby improving network performance in various visual tasks.

The Dense Convolutional Network (DenseNet) proposed by Huang et al. [32] connects each layer to every other layer in a feedforward manner to alleviate the vanishing gradient problem and improve feature utilization. The densely connected Siamese network SNUNet-CD, proposed by Fang et al. [33], enhances feature extraction across different semantic levels through compact information transfer between the encoder and decoder and the integration of a channel attention module (ECAM), improving change detection accuracy. The Multi-scale Densely Connected U-Net (MDU-Net) by Zhang et al. [34] establishes multi-scale dense connections between the encoder and decoder, which strengthens feature propagation by fusing feature maps from different levels. Additionally, it introduces quantization techniques to mitigate overfitting, thereby enhancing biomedical image segmentation performance. Ju et al. [35] proposed an Interval Dense Connection Strategy applied to the Swin Transformer, creating a new super-resolution model called SwinOIR. By improving feature reuse, it achieved optimal image super-resolution performance on multiple benchmark datasets.

Dense connections address issues like inefficient feature reuse and the vanishing gradient problem by enhancing information flow and ensuring robust gradient transmission. We innovatively apply these connections to improve small object detection accuracy.

## 3. Methods

In this section, we propose a novel detection framework, named the YOLO-DHGC algorithm, to address the challenges of low localization accuracy and difficulty in extracting effective features for small object detection. The algorithm employs a two-stream structure based on an edge-gated branch for feature extraction and integrates DenseHRNet as the backbone in the conventional detection stream. This approach captures rich detail and boundary information of small objects effectively.

### 3.1. YOLO-DHGC

**Figure 1:**
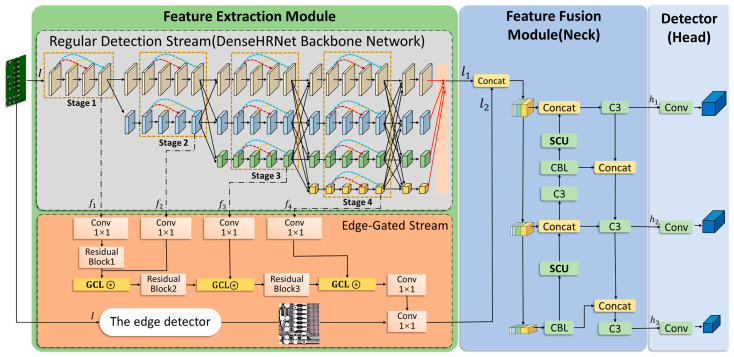
Framework of the YOLO-DHGC algorithm.

YOLO, because of its efficiency in real-time object detection, excels in various applications. Its basic structure includes three main parts: the backbone, the neck, and the head. The backbone is responsible for extracting features from the input image. The neck performs feature fusion, often including components like the Feature Pyramid Network (FPN). The head is responsible for the final bounding box regression and class prediction. Based on this, we have innovatively enhanced the framework to better detect small objects in complex environments. The overall framework of the small object detection algorithm YOLO-DHGC is shown in Figure 1. The main structure is divided into feature extraction module, feature fusion module, and detection head.

The feature extraction module uses a two-stream structure, consisting of a regular detection stream and an edge-gated stream. The regular detection stream employs the DenseHRNet backbone network. This backbone network employs a four-stage architecture with dense connections, combining high-resolution feature extraction and multi-resolution fusion. It enhances small object detection by preserving detailed features across different scales. The edge-gated stream uses gated convolutional layers to facilitate the transfer of information from the regular detection stream. This process filters out redundant information and highlights boundary details. In the feature fusion module, outputs from both streams are combined through channel merging. The module integrates features from different levels of the backbone network, each with varying receptive fields and semantic levels. This process generates feature maps at three different scales for the classification and regression prediction of small, medium, and large objects in the detection head.

### 3.2. Feature Extraction Module

To reduce the impact of interfering information, this paper proposes a two-stream structure in feature extraction, illustrated in Figure 1. The regular detection stream uses DenseHRNet as its backbone, which employs a dense connection mechanism to enhance feature reuse and transfer in the high-resolution HRNet, capturing multi-scale contextual information with different receptive fields. The edge-gated stream filters out irrelevant information through gated convolutional layers, focusing on processing shape boundary information, and incorporates edge detection to enhance edge information learning. Finally, the outputs of the two streams are combined in the feature fusion structure to improve the detection accuracy of small objects.

#### 3.2.1. Regular Detection Stream with DenseHRNet Backbone Network

To overcome the loss of small object information caused by multi-layer downsampling in traditional YOLO networks, this paper designs the DenseHRNet backbone network. DenseHRNet combines the high-resolution feature extraction capability of HRNet [36] with the dense connection mechanism of DenseNet [37]. This improvement enhances the transfer and fusion of shallow and deep features while maintaining the integrity of high-resolution feature maps, ensuring the preservation of small object details. Thereby, the accuracy of small object detection is improved.

First, we introduced a dense connection mechanism to ensure feature reuse, effectively alleviating the vanishing gradient problem and enabling the network to extract and transmit feature information across multiple layers. Second, in the forward convolution process, DenseHRNet incorporates a high-resolution feature map main branch, ensuring the network can capture rich detail information of small targets, thereby enhancing its ability to recognize them.

**Figure 2:**
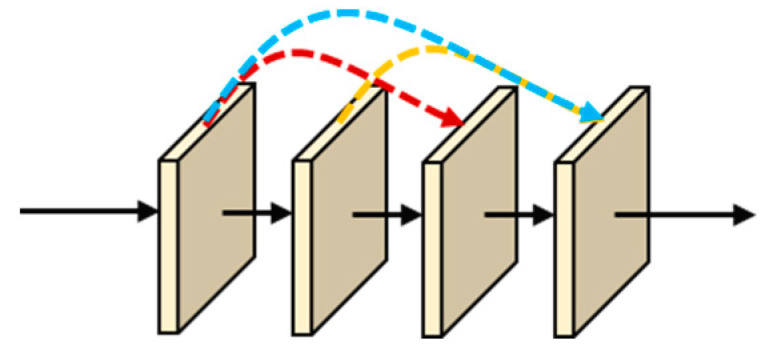
Single-resolution DenseHRNet connection. Different colored arrows represent the flow of information from different layers.

The connection method of the single-resolution branch DenseHRNet, which incorporates dense connections, is shown in Figure 2. If the depth of a resolution branch is l layers, then the dense connection establishes l(l+1)/2 connections. The output $x_{l}$ of the lth layer can be expressed as follows:

(1) $$x_{l}=H_{l}\left(\left[\begin{array}{c} x_{0},x_{1},x_{2},\cdots ,x_{l-1} \end{array}\right]\right)$$

where $\left[\begin{array}{c} x_{0},x_{1},x_{2},\cdots ,x_{l-1} \end{array}\right]$ represents channel concatenation. The input of the lth layer of the network contains all the outputs of layers 1, 2, 3, ......, l−1. $H_{l}(x)$ denotes the set of nonlinear transformation functions, including batch normalization, nonlinear activation function ReLU, pooling, and convolution operations.

**Figure 3:**
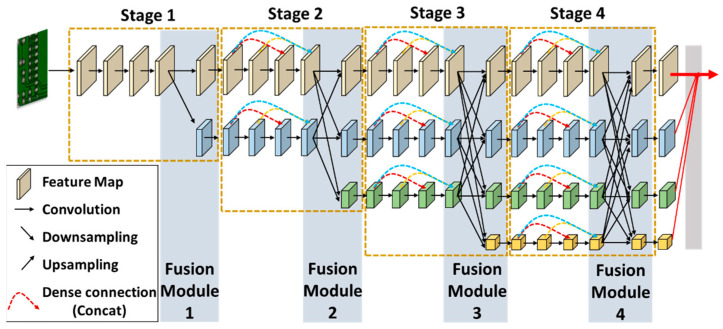
The architecture of DenseHRNet backbone network. The black arrows represent information directly passed from the previous layer, while the other colored arrows indicate information passed across different layers. These colored arrows illustrate the dense connection mechanism, where each layer is directly connected to all subsequent layers, enhancing feature reuse and information flow.

In accordance with the DenseHRNet single-resolution branching implementation method, this paper constructs four branches with different resolutions by downsampling the high-resolution branch three times. This approach extracts four small object features with varying resolutions. A dense connection mechanism is added to each parallel branch from the second to the fourth stage in the original HRNet, creating the DenseHRNet backbone network structure, illustrated in Figure 3. In the fusion modules 2, 3, and 4, each parallel branch first performs batch normalization, followed by ReLU activation. It then reduces the output feature map channels by half using 1 × 1 convolution. Finally, it completes the feature fusion operations, including upsampling and downsampling.

**Figure 4:**
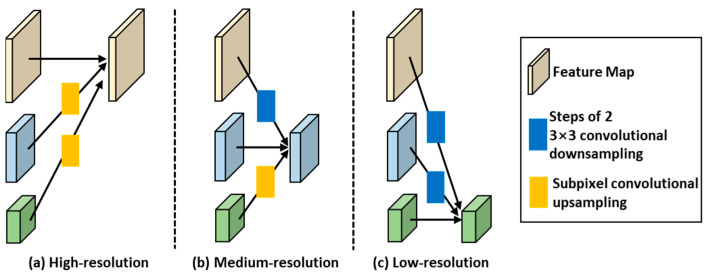
Fusion process for different resolution feature maps.

In DenseHRNet, the fusion process of different resolution feature maps is illustrated in Figure 4. Within the fusion module, feature maps of the same resolution are directly copied to the next layer. Low-resolution feature maps are upsampled to obtain high-resolution feature maps, and then their channels are matched using a 1 × 1 convolutional layer. High-resolution feature maps are downsampled to obtain low-resolution feature maps, and a 3 × 3 convolutional kernel with a step size of 2 is used for this downsampling in the fusion module. Since feature maps suffer from information loss during downsampling, the fusion module does not use maximum pooling or combined pooling operations to reduce the information loss during downsampling. After completing the upsampling and downsampling processes, the feature maps with different resolutions are fused through corresponding element-wise addition.

Due to the dense connection mechanism, DenseHRNet leverages intermediate sub-layer features. This means the final features not only integrate characteristics from different levels but also aggregate all previous layers’ features across various scales. The feature maps learned by any layer of DenseHRNet can be accessed by the last layer. When the gradient is back-propagated, some information reaches each intermediate layer directly, bypassing the deeper layers. This forces intermediate layers to learn more discriminative features, facilitating training and improving network performance. Consequently, the proposed DenseHRNet network combines the advantages of shallow and deep features, resulting in richer feature maps that provide a clearer representation of small object features while retaining sufficient local details.

#### 3.2.2. Edge-Gated Stream

In constructing the edge-gated flow, this paper innovatively designs a two-input architecture aimed at capturing and enhancing object boundary information from two different perspectives. This design not only utilizes the multi-level feature maps extracted by the DenseHRNet backbone but also incorporates raw image gradients from an edge detector as an independent input source. We innovatively introduce gradient information obtained through edge operators into the two-stream structure, specifically using the Canny operator in our experiments. This unique two-input strategy enables the network to simultaneously learn implicit features from the deep learning model and explicit edge cues. Specifically, the edge operator accurately locates edges in the image, which are often overlooked or weakened in traditional convolutional neural networks. We combine these gradient maps with the feature maps from different stages from DenseHRNet, dynamically adjusting feature weights at each layer through a gating mechanism, thereby achieving high sensitivity and optimization of edge details.

**Figure 5:**
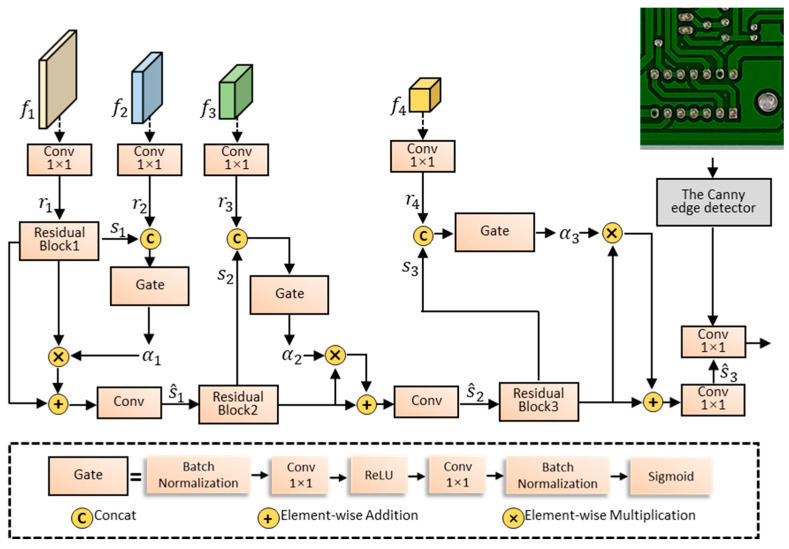
Structure of the edge-gated stream.

The overall structure of the edge-gated stream is shown in Figure 5, where three gated convolutional layers are used. These layers are crucial in the edge-gated stream because they effectively filter out irrelevant information. This capability allows the stream to suppress non-edge-related information, ensuring that only edge-related feature information is processed. The input of the gated convolutional layer comes from two parts: one is the feature map $f_{t}$ from this level, which belongs to the low-level features, and the purpose is to filter the irrelevant feature information to obtain the edge feature information. The other is the feature map $f_{t+1}$ from the higher level, which is a higher-level semantic understanding of the image and guides the network to extract more complete edge-related features. For each feature map $f_{t}$ from the shallow layer, a 1 × 1 convolution is performed to generate a regular detection stream feature map $r_{t}$. $r_{t+1}$ with high-level semantic information are aggregated with the boundary feature map $s_{t}$, which passes through the gating unit to generate the attention feature map $\alpha_{t}\in R^{1\times H\times W}$. In particular, $r_{1}$ passes through the residual structure to obtain the boundary feature map $s_{1}\in R^{C\times H\times W}$ for the C channel, as shown in Equation (2).

(2) $$\alpha_{t}=\sigma (C_{1\times 1}(s_{t}∥r_{t}))$$

where ∥ represents the channel-merging calculation of the feature map. $\alpha_{t}\;$ denotes the weight of each pixel in the feature map, with higher weights assigned to regions with significant boundary information. Each element in $\;\alpha_{t}$ is within the range (0, 1). The larger the pixel value in the attention feature map, the more important the corresponding feature in $s_{t}$ is.

The other part of $s_{t}$ will be added element-wise with the features $\alpha_{t}$ processed by the gated unit through skip connections, resulting in the final output-fused feature ${\hat{s}}_{t}\in R^{C\times H\times W}$, as shown in Equation (3). After passing through the residual structure, ${\hat{s}}_{t}$ yields the boundary feature map $s_{t+1}$, which serves as the input for the t+1 gated convolution module. The semantic features $r_{t+2}$ from higher layers guide and constrain this filtering process, ensuring the gating mechanism retains only edge-related feature information. Finally, the image gradient information is fused with the feature maps output by the gated convolution layer through channel concatenation, resulting in more refined edge features. This serves as the output of the shape stream branch, aiding in the learning of small objects.

(3) $${\hat{s}}_{t}^{\left(i,j\right)}={\left(s_{t}\;Ⓖ\;w_{t}\right)}_{\;(i,j)}={\left(\left(s_{t_{\left(i,j\right)}}⊙\alpha_{t_{\left(i,j\right)}}\right)+s_{t_{\left(i,j\right)}}\right)}^{T}w_{t}$$

where Ⓖ represents the gated convolution calculation, ⊙ denotes the element-wise multiplication operation, and (i,j) indicates the pixel position.

#### 3.2.3. Two-Stream Object Detection Structure Based on Edge-Gated Branch

In the two-stream object detection structure based on the edge-gated branch, the regular detection stream and edge-gated stream collaborate with each other. The regular detection stream extracts features using the DenseHRNet backbone network, generating more features and achieving a higher semantic understanding of the image, which aids in accurately identifying small objects. The gated convolutional layer helps the edge-gated stream focus only on edge-related parts from the beginning. It uses features extracted by the regular detection stream, highlighting boundary information in the network. This assists in the accurate localization of small objects.

### 3.3. Feature Fusion Module

In this paper, the Feature Pyramid Network (FPN) is used in combination with the Path Aggregation Network (PAN) to fuse the above multi-scale features. The FPN realizes information sharing among feature maps through up-down paths and lateral connections. Specifically, starting with high-level feature maps, upsampling operations are used to fuse them with low-level feature maps. This forms a set of feature maps with multi-scale characteristics. This process can be described in Equation (4):

(4) $$F_{FPN}^{l}=Conv\left(F_{FPN}^{l-1}+UpSample\left(F_{high}^{l}\right)\right)$$

where $F_{FPN}^{l}$ denotes the FPN feature map of the *l*th layer, $F_{FPN}^{l-1}$ is the FPN feature map of the previous layer, and $F_{high}^{l}$ is the feature map from the deeper layer. Through this level-by-level fusion, each layer of FPN feature maps contains multi-level semantic information.

The PAN further optimizes the propagation path of the features through the bottom-up path so that the low-level features can flow upward and be fused with the high-level features in a secondary way, as shown in Equation (5):

(5) $$F_{PAN}^{l}=Conv\left(F_{FPN}^{l}+DownSample\left(F_{PAN}^{l+1}\right)\right)$$

Here, $F_{PAN}^{l}$ denotes the PAN feature map of the *l*th level, while $F_{PAN}^{l+1}$ is the PAN feature map of the next level. Through the down-up path of the PAN, the rich details of the low-level features are preserved and combined with the semantic information of the high-level features to form a more comprehensive feature representation.

Through the organic combination of the FPN and PAN, features at different scales are effectively extracted and fused. This provides strong support for the subsequent detection head, leading to excellent performance in the object detection task.

### 3.4. Object Detection Heads

The detection head’s main task is to classify objects and perform bounding box regression on the feature maps from previous modules. To achieve this, the loss function is crucial for guiding the training process. It provides necessary gradient information through backpropagation, optimizing the model. The accuracy and reliability of the detection head’s output are measured and optimized using three synergistic loss functions: classification loss, confidence loss, and bounding box regression loss.

In this paper, binary cross-entropy (BCE) loss quantifies the difference between the predicted and actual categories for categorization. For each prediction frame, the model outputs a series of probability distributions corresponding to the likelihood of each category. Assuming that the predicted probability is $p_{i}$ and the true label is $t_{i}$, the binary cross-entropy loss for category i can be expressed as follows:

(6) $$L_{cls}=-t_{i}log\left(p_{i}\right)-\left(1-t_{i}\right)log-\left(1-p_{i}\right)$$

This loss function encourages the model to converge to a prediction probability of 1 for positive categories and 0 for negative categories, thus improving classification accuracy.

Confidence loss assesses the confidence of a model about whether there is an object in a given prediction frame and whether the prediction frame is accurate. In this paper, the BCE loss is used as the confidence loss. If the prediction box contains an object and the IoU of the box to the true box is above a certain threshold, the confidence object $c_{i}$ is set to 1; otherwise, the confidence object is set to 0. For the confidence prediction ${\hat{c}}_{i}$ for each prediction box, the confidence loss can be expressed as follows:

(7) $$L_{conf}=-c_{i}log-\left({\hat{c}}_{i}\right)-\left(1-c_{i}\right)\log\left(1-{\hat{c}}_{i}\right)$$

By minimizing the loss of confidence, the model is trained to distinguish which prediction frames actually contain the object and which frames are background or inaccurate.

The bounding box regression loss assesses the positional deviation between the predicted and actual boxes. In this paper, CIoU loss (Complete Intersection over Union loss) is adopted as the bounding box regression loss. Assuming that $b_{pred}$ is the predicted frame and $b_{true}$ is the true frame, the CIoU loss can be expressed as follows:

(8) $$L_{bbox}=1-CIoU\left(b_{pred},b_{true}\right)$$

where $CIoU\left(b_{pred},b_{true}\right)$ consists of the IoU part, the centroid distance penalty $\rho \left(c_{pred},c_{true}\right)$, and the aspect ratio consistency penalty v.

(9) $$IoU=\frac{\left|b_{pred}\cap b_{true}\right|}{\left|b_{pred}\cup b_{true}\right|}$$

(10) $$\rho \left(c_{pred},c_{true}\right)=\frac{∥c_{pred}-c_{true}{∥}^{2}}{c^{2}}$$

(11) $$v=\frac{4}{\pi^{2}}{\left(arctan\frac{w_{pred}}{h_{pred}}-arctan\frac{w_{true}}{h_{true}}\right)}^{2}$$

where $c_{pred}$ is the centroid of the prediction box, and $c_{true}$ is the centroid of the real box. c denotes the diagonal length of the smallest outer rectangle encompassing both the prediction and real boxes. The variables w and h represent the box’s width and height, respectively.

The CIoU loss function combines these three components and significantly improves the localization accuracy of the detection model by optimizing the location, size, and shape of the bounding box.

## 4. Experiments

### 4.1. Dataset

#### 4.1.1. PKU-Market-PCB Dataset

In this paper, the effectiveness of the proposed algorithm is verified using the PKU-Market-PCB small object defect public dataset. The dataset is provided by the Intelligent Robotics Open Laboratory at Peking University. The original dataset contains a total of 693 images with six types of defects. Each image has at least three defects, the smallest being about 10 × 15 pixels, accounting for approximately 0.0031% of the image area. This makes it suitable for examining the performance of small object detection algorithms.

The dataset is split into a training set, validation set, and test set in an 8:1:1 ratio. This results in 555 training images, 69 validation images, and 69 test images. Due to the small number of samples in the original dataset, issues like low detection accuracy, poor robustness, and overfitting may occur during training. Therefore, it is necessary to expand the dataset to increase data diversity. The 555 images in the training set undergo data enhancement operations such as random rotation, random cropping, brightness adjustment, and noise addition. After enhancement, the total number of training images is 3330. Information about the enhanced dataset is shown in Table 1.

#### 4.1.2. NEU-DET Hot Rolled Steel Surface Defect Dataset

In addition, this paper uses the NEU-DET public dataset of hot-rolled steel surface defects to validate the effectiveness of the algorithm. The NEU-DET dataset, released by Northeastern University, consists of 1,800 grayscale images with six types of defects. Each type contains 300 images. The smallest defects are about 20 × 20 pixels, approximately 0.01% of the image area. This makes the dataset suitable for examining the performance of small object detection algorithms.

The dataset is split into training, validation, and test sets in an 8:1:1 ratio. This results in 1440 training images, 180 validation images, and 180 test images. The detailed label distribution is shown in Table 2.

#### 4.1.3. TinyPerson Dataset

In this paper, the TinyPerson dataset is used to test the detection effect of the designed small object detection algorithm in areas other than industry and to verify the robustness and generalizability of the algorithm. TinyPerson is a dataset specially designed for the detection of small objects at long distances and against large backgrounds. It contains 1610 labeled images, with 85% of the objects having a resolution of less than 20 × 20 pixels. This accounts for about 0.00004% of the image area, making it suitable for testing the performance of small object detection algorithms. The dataset provider has divided it into 794 training images and 816 validation images, totaling 72,651 manually labeled instances. The dataset information is shown in Table 3.

#### 4.1.4. Self-Constructed Backlight Panel Micro-Defect Dataset

Publicly available datasets suitable for small object detection in industry are scarce, and high-quality datasets are often the basis for deep learning model training. To test the effectiveness of this algorithm in practical applications, this paper collaborates with an industrial vision technology R&D company to construct a dataset of small defects on backlight panel surfaces. The dataset contains 303 images and is divided into a training set, validation set, and test set in a ratio of 8:1:1. Due to the limited number of images, data enhancement is performed on 241 images in the training set to expand the dataset and increase the model’s generalization ability. After enhancement, the training set contains 3374 images. Information about the enhanced dataset is shown in Table 4.

### 4.2. Assessment of Indicators

In order to comprehensively evaluate the model’s performance, this study adopts a series of metrics widely recognized in the field of deep learning, covering precision (*P*), recall (*R*), average precision (*AP*), and mean average precision (*mAP*). The specific definitions of each indicator are as follows:

Precision (*P*): a measure of the proportion of positive samples correctly predicted by the model out of all its predicted positive samples, i.e., the ratio of true positives (*TP*s) to true positives plus false positives (FPs).

(12) $$P=\frac{TP}{TP+FP}$$

Recall (*R*): reflects the proportion of actual positive samples identified by the model out of all actual positive samples, i.e., true positives (*TP*s) divided by true positives plus false negatives (*FN*s).

(13) $$R=\frac{TP}{TP+FN}$$

Average precision (*AP*): for a single category, a composite metric based on the average of the interpolated precision at different recall levels is provided to assess the model’s ability to detect in that category.

(14) $$AP=\sum_{i=1}^{n-1}\left(r_{i+1}-r_{i}\right)p_{interp}\left(r_{i+1}\right)$$

where $r_{i}$ denotes the recall value corresponding to the first interpolation at the first interpolation of the precision interpolation segment in ascending order.

Mean average precision (*mAP*): as an average of *AP*s across all categories, *mAP* provides a global view of the model’s overall detection performance. It is a particularly critical evaluation criterion for the object detection task.

(15) $$mAP=\frac{\sum_{i=1}^{K}AP_{i}}{K}$$

where K is the category of the object.

In the calculation process, the Intersection over Union (IoU) threshold is usually set to 0.5, that is, mAP@0.5, to assess the effectiveness of the prediction box. In addition, mAP@0.5 0.95 comprehensively considers the performance of IoU within the range of 0.5 to 0.95 (with an interval of 0.05), further enhancing the comprehensiveness of the evaluation. AP_S_, AP_M_, and AP_L_ are the average precision for small-, medium-, and large-sized objects, specifically for objects with pixel areas less than 32 × 32, between 32 × 32 and 96 × 96, and greater than 96 × 96. In subsequent experiments, mAP@0.5, mAP@0.75, mAP@0.5, AP_S_, AP_M_, and AP_L_ evaluation models’ accuracy will be comprehensively used on the PCB dataset, NEU-DET dataset, TinyPerson dataset, and backlight panel dataset.

Since the absolute scale of the object in the TinyPerson dataset is too small, its authors divide the scale size into three major categories: tiny (2, 200), small (20, 32), and all (2, inf). The tiny category is further divided into three small objects: tiny1 (2, 8), tiny2 (8, 12), and tiny3 (12, 20). Due to the small size of objects, position detection is challenging. Therefore, the IoU confidence threshold options are 0.25, 0.5, and 0.75. The evaluation indexes for single-category average accuracy include $AP_{0.5}^{tiny1}$, $AP_{0.5}^{tiny2}$, $AP_{0.5}^{tiny3},$ $AP_{0.5}^{tiny},$ $AP_{0.25}^{tiny},$ $AP_{0.75}^{tiny},and\;AP_{0.5}^{small}$.

### 4.3. Experimental Configurations

The experimental results are affected by the performance of hardware devices, software versions, and differences in training platforms. The detection performance of models trained on different devices will vary. The equipment used in the experiments included an Intel^®^ Xeon^®^ Platinum 8163 CPU and a GeForce RTX 2080 Ti 11G GPU. The operating system was Ubuntu 18.04, with Python 3.7 as the programming language. The deep learning framework was PyTorch, using CUDA version 10.1 and CuDNN version 7.6.5.

The algorithms were retrained in the training process and were not loaded with pre-training weights. The neural network model optimizers were all set to SGD with a learning rate of 0.01 and a decay of 0.0005. The input image sizes were resized to 640 × 640, and the batch size was set to 16. A total of 300 training rounds were conducted until the loss function converges.

### 4.4. Benchmark Selection Comparison Experiment

To accurately select the optimal base model for a specific application scenario, this study focused on two representative versions of the YOLO series algorithms: YOLOv5 and YOLOv8. An in-depth comparison experiment was conducted. Since YOLOv5 and YOLOv8 are high-performance and widely used versions, integrating key optimizations and technological advances, directly comparing them efficiently identifies the most suitable model for a given task. This approach eliminates the need to evaluate each version individually and ensures the research focuses on the most competitive iterations. In this paper, we selected the appropriate algorithm for improvement by evaluating it on both the PCB and NEU-DET datasets. The experimental results are shown in Table 5.

Based on the data in Table 5, we can see that YOLOv5 performs better than YOLOv8 on the industrial defect recognition task. YOLOv5 adopts an anchor frame design that provides prior information to help the model better capture features of small objects, especially when recognizing small defects in complex backgrounds, resulting in higher accuracy. It can adapt to small objects of different sizes by adjusting the anchor frame size and scale. Additionally, YOLOv5 is relatively lightweight, allowing it to run more efficiently for small object detection tasks. Therefore, this paper focused on improving the small object detection algorithm based on YOLOv5.

### 4.5. Model Training

**Figure 6:**
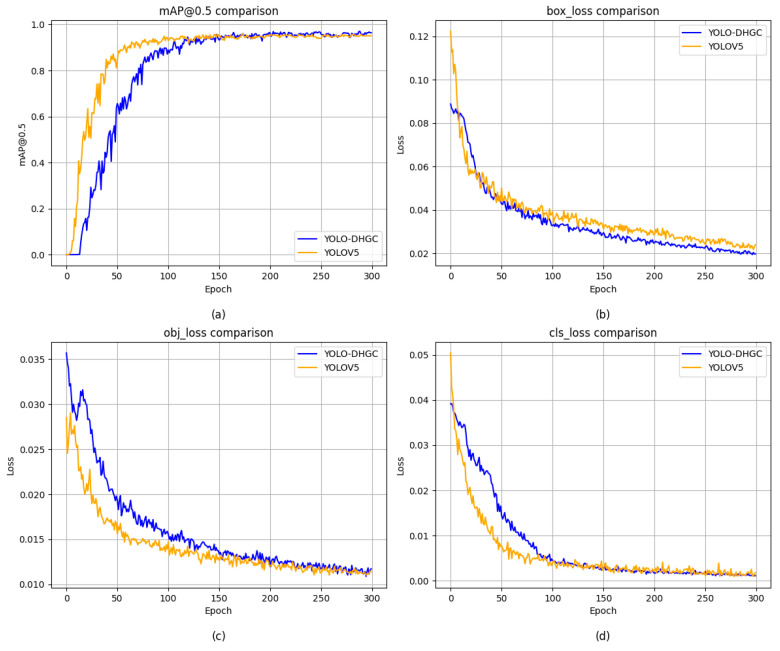
Comparison of mAP@0.5 and loss change curves during training. (**a**) represents a comparison in mAP@0.5; (**b**) represents a comparison in bounding box regression loss; (**c**) represents a comparison of confidence loss; (**d**) represents a comparison in classification loss.

To illustrate the training dynamics of the proposed YOLO-DHGC model, the mAP@0.5 change curves and loss change curves during the training process are provided (as shown in Figure 6). These curves offer insights into how the model’s performance evolves over epochs and how effectively it minimizes prediction errors.

As depicted in Figure 6, the mAP@0.5 metric for YOLO-DHGC demonstrates a steady increase with each epoch, surpassing YOLOv5’s performance. This trend suggests that YOLO-DHGC is more efficient at learning discriminative features from the dataset. Moreover, the loss components—box_loss, obj_loss, and clc_loss—all exhibit a downward trend for YOLO-DHGC, indicating that the model is improving its localization, confidence scoring, and class prediction abilities, respectively.

The rapid decline in losses and the superior mAP@0.5 scores throughout the epochs highlight the effectiveness of the proposed architecture in terms of both learning speed and accuracy.

### 4.6. Experimental Results

#### 4.6.1. PKU-Market-PCB Dataset

To objectively verify the performance of the YOLO-DHGC algorithm designed in this paper, it was compared with single-stage detection algorithms SSD, RetinaNet, YOLOv4, YOLOv5, YOLOX, and YOLOv7, as well as two-stage detection algorithms Faster R-CNN and Cascade R-CNN, all within the same configuration environment. Additionally, a Transformer-based object detection algorithm, DETR, was included for comparison. The detection accuracy of different algorithms on the PKU-Market-PCB dataset is shown in Table 6.

Comparative experimental results on the PKU-Market-PCB dataset show that the YOLO-DHGC algorithm proposed in this paper achieved the best results in all accuracy metrics compared to several mainstream models. YOLO-DHGC’s mAP@0.5, mAP@0.75, and mAP@0.5:0.95 achieved 96.3%, 48.5%, and 54.0%, respectively, which were 2.0%, 0.7%, and 1.4% higher than YOLOv5s. The small object detection accuracy of AP_S_ reached 48.5%, showing a 2.6% improvement over YOLOv5s. In addition, the AP_L_ of large object detection accuracy of YOLO-DHGC reached 56.3%, a slight improvement of 0.4% compared to YOLOv5. Compared with YOLOv7s, YOLO-DHGC’s mAP@0.5, mAP@0.75, and mAP@0.5:0.95 increased by 0.5%, 0.3%, and 0.7%, respectively, and the accuracy of small object detection AP_S_ increased by 1.9%. Compared to DETR, YOLO-DHGC improved mAP@0.5, mAP@0.75, and mAP@0.5:0.95 by 1.1%, 4.1%, and 4.8%, respectively, demonstrating superior detection performance.

Table 7 shows the detection accuracy of each algorithm for six different defects on the PKU-Market-PCB dataset. The mAP@0.5 obtained by YOLO-DHGC for six types of defects—Missing Hole, Mouse Bite, Open Circuit, Short, Spur, and Spurious Copper—were 99.3%, 97.4%, 95.9%, 97.2%, 91.8%, and 96.1%, respectively. These results were 2.9%, 2.5%, 3.8%, 1.3%, 1.1%, and 0.6% higher than the baseline method YOLOv5s, showing a significant overall accuracy improvement. Among the compared algorithms, YOLOv7s achieved the highest detection accuracy for Short defects. However, YOLO-DHGC achieved the highest mAP@0.5 accuracy for the other five defect types. Therefore, compared to mainstream object detection algorithms, the YOLO-DHGC algorithm has advantages in detecting small PCB defects.

The substantial improvement of the YOLO-DHGC algorithm in PCB small-defect detection accuracy proves that the improvement strategy for small object detection in this paper is effective. However, the experiments found that the accuracy of large object detection with YOLO-DHGC is not significantly improved. This may be because the improved design enhances the response of shallow features to small objects, reducing the importance of high-level features. As a result, the model focuses too much on fine details and overlooks the overall image information, affecting large-scale object detection.

**Figure 7:**
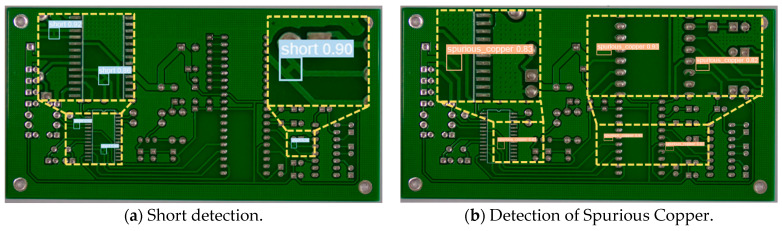
YOLO-DHGC visualization results for Short and Spurious Copper defects.

Figure 7 shows the visualization results of YOLO-DHGC for PCB defect detection. It can be seen that the proposed algorithm detects both Short and Spurious Copper defects completely, with no misdetections or omissions.

**Figure 8:**
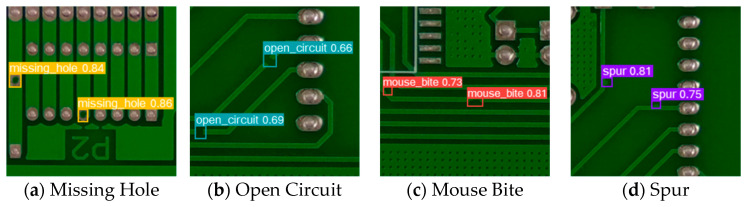
Visualization of detection results of YOLO-DHGC on PKU-Market-PCB dataset.

Figure 8 shows the actual detection results of YOLO-DHGC. The detected defect types included Missing Hole, Open Circuit, Mouse Bite, and Spur. To clarify the detection results, all images were locally enlarged. Each detected defect was labeled with a confidence score on its detection box. A higher score indicates a greater likelihood of the defect’s presence at that location. YOLO-DHGC detected all defects completely and with high confidence, achieving the highest confidence level of 0.86 for vulnerability defects. Moreover, it is worth noting that the images may contain multiple types of defects simultaneously, such as Missing Hole and Open Circuit within the same image. Despite the complexity introduced by the presence of multiple defects, the YOLO-DHGC algorithm demonstrated its capability to accurately identify and localize each defect individually with high precision. This indicates that the YOLO-DHGC algorithm exhibits superior overall performance in both localization and classification of small objects.

#### 4.6.2. Analysis of Experimental Results of NEU-DET Hot-Rolled Steel Surface Defect Dataset

To evaluate the detection performance of YOLO-DHGC on the NEU-DET hot-rolled steel surface defect dataset, the designed YOLO-DHGC was compared with various mainstream algorithms under the same environment configuration. The detection accuracies of different algorithms on the NEU-DET dataset are shown in Table 8.

The YOLO-DHGC algorithm proposed in this paper achieved the best results in all accuracy indicators except AP_L_, verifying its effectiveness in detecting small defects on hot-rolled steel surfaces. YOLO-DHGC’s mAP@0.5, mAP@0.75, and mAP@0.5:0.95 were 81.6%, 47.8%, and 50.2%, respectively. These were 4.7%, 1.6%, and 3.0% higher than the baseline method YOLOv5s. The AP_S_ for small object detection reached 44.3%, which is 2.8% higher than YOLOv5s, showing a significant improvement in small object detection accuracy. The AP_L_ for large object detection was 51.2%, on par with YOLOv5. Overall, YOLO-DHGC outperforms YOLOv5s in all accuracy metrics except AP_L_. Compared to YOLOv7s, the mAP@0.5, mAP@0.75, and small object detection accuracy of AP_S_ improved by 1.4%, 0.7%, and 1.2%, respectively. However, the AP_L_ for large object detection was 0.6% lower than YOLOv7s.

Table 9 shows the detection accuracy for six different defects on the NEU-DET dataset. YOLO-DHGC achieved mAP@0.5 results of 51.1% for Crazing, 85.8% for Inclusion, 91.5% for Patches, 87.9% for Pitted Surface, 78.9% for Rolled-in Scale, and 87.4% for Scratches. Compared to YOLOv5s, detection accuracy improved by 2.8%, 3.0%, 2.1%, 2.0%, 3.7%, and 3.5%, respectively. Among the compared algorithms, YOLOv7s had the highest detection accuracy for Inclusion defects at 86.5%. YOLOX performed best for Rolled-in Scale defects with an accuracy of 80.0%. For the other four defects, YOLO-DHGC achieved the highest mAP@0.5 accuracy. Therefore, compared to mainstream detection algorithms, the YOLO-DHGC algorithm shows a strong detection effect on surface defects of hot-rolled steel.

**Figure 9:**
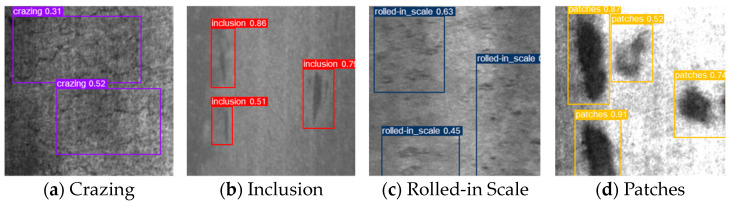
Comparison of visual results of YOLO-DHGC detection on NEU-DET dataset.

Figure 9 shows the visualization results of the actual detection by YOLO-DHGC. The detected defects included Cracking, Inclusion, Rolled-in Scale, and Scratches. YOLO-DHGC successfully detected all defects with high confidence, achieving the highest confidence of 0.87 when detecting plaque defects. This demonstrates that the YOLO-DHGC algorithm has superior performance in locating and classifying small object defects. In summary, YOLO-DHGC has a comprehensive ability to detect small defects on the surface of hot-rolled steel.

#### 4.6.3. Analysis of Experimental Results on TinyPerson Dataset

To demonstrate that the YOLO-DHGC algorithm is effective for detecting small objects beyond industrial scenes, and to show its robustness and generalization, this paper used the TinyPerson dataset. YOLO-DHGC was compared with mainstream object detection algorithms, and the experimental results are shown in Table 10. The detection object pixels are categorized as follows: tiny (2, 20), tiny1 (2, 8), tiny2 (8, 12), tiny3 (12, 20), small (20, 32). The metrics $AP_{0.25}^{tiny},\;AP_{0.5}^{tiny},$ and $AP_{0.75}^{tiny}$ indicate the average accuracy of the tiny object when the preset IoU threshold is 0.25, 0.5, and 0.75, respectively. $AP_{0.5}^{\mathrm{small}}$ indicates the average accuracy of the small object at a preset IoU threshold of 0.5.

When the object scale of YOLO-DHGC was (2, 20) pixels and (20, 32) pixels, the detection accuracy of $AP_{0.5}^{tiny}$ and $AP_{0.5}^{small}$ reached 53.98% and 67.92%, respectively. This was 1.53% and 2.58% higher than the baseline algorithm YOLOv5s. Compared to YOLOv7s, the increase was 1.31% and 1.57%. For objects with a scale of (2, 20) pixels, the detection accuracy of YOLO-DHGC for objects with (2, 8) pixels, (8, 12) pixels, and (12, 20) pixels was 38.43%, 58.91%, and 64.23%. This was 2.86%, 4.68%, and 3.21% higher than YOLOv5s, and compared to YOLOv7s, it increased by 2.74%, 3.24%, and 2.10%. The small object detection algorithm designed in this paper comprehensively achieved better detection accuracy than mainstream object detection algorithms on the TinyPerson dataset. This proves that the YOLO-DHGC algorithm significantly improved the detection performance of small objects and achieved good results beyond industrial scenes.

Based on the experimental results, although detection algorithms like Faster R-CNN and YOLOv7 perform well on general object datasets such as MS COCO or PASCAL VOC, they do not achieve good results on small object datasets. YOLOv7 achieves better detection results than YOLOv5 on the COCO dataset, but its accuracy on the TinyPerson dataset is not significantly different from YOLOv5. This is likely because these algorithms are designed for conventional object scales, and the TinyPerson dataset’s object scale is too small, affecting performance.

**Figure 10:**
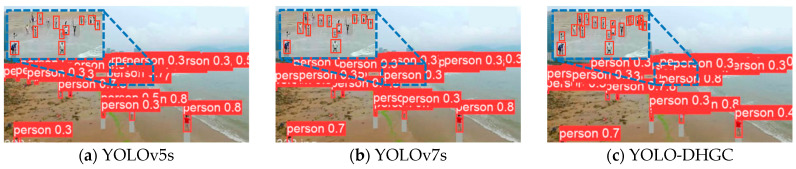
Comparison of algorithm detection results on TinyPerson dataset.

Figure 10 shows the actual detection results of the YOLO-DHGC, YOLOv5s, and YOLOv7s algorithms on the TinyPerson dataset. The results indicate that YOLOv5s missed a large number of small objects, with low detection confidence. YOLOv7s also missed some very small objects. Compared to YOLOv5s and YOLOv7s, YOLO-DHGC detected more small-scale people, classified and located dense crowds better, and had a high confidence level. This indicates that the improvement strategy designed in this paper effectively enhances the algorithm’s detection performance on small objects, not only in industrial settings but also for various small objects in everyday life.

#### 4.6.4. Self-Constructed Backlight Panel Micro-Defect Dataset

To address the low accuracy of small-defect detection in real industrial scenarios, this paper used a self-built backlight panel small-defect dataset. The YOLO-DHGC algorithm was trained to test its ability to detect small defects on backlight panels. Comparison experiments were conducted with mainstream algorithms in the same configuration environment. The detection accuracies of different algorithms on the self-constructed backlight panel micro-defect dataset are shown in Table 11.

On the self-built backlight panel tiny-defects dataset, the YOLO-DHGC algorithm proposed in this paper achieved the best results in all accuracy indexes compared to mainstream algorithms. This verifies its advantage in detecting tiny defects on backlight panel surfaces. YOLO-DHGC’s mAP@0.5 and mAP@0.5:0.95 were 93.4% and 52.8%, respectively. These were 10.2% and 9.6% higher than the baseline method YOLOv5s. The accuracy of small object detection (AP_S_) reached 47.4%, showing a significant improvement of 9.2% compared to YOLOv5s. Compared to YOLOv7s, YOLO-DHGC’s mAP@0.5 and mAP@0.5:0.95 increased by 4.1% and 2.9%, respectively, and the AP_S_ increased by 3.3%.

Table 12 shows the accuracy for five types of defects on the self-built backlight panel small-defect dataset. YOLO-DHGC achieved improvements in mAP@0.5 for White_spot, Black_spot, Scratching, Black_mass, and White_mass. The accuracy of YOLOv5s was significantly improved by 5.2%, 15.2%, 11.8%, 22.3%, and 4.5%, with final accuracies of 90.4%, 79.3%, 96.4%, 98.2%, and 98.9%, respectively.

The sizes of the three types of defects—Scratching, Black_mass, and White_mass—are larger compared to White_spot and Black_spot. This allows these three types to achieve a higher accuracy rate. Black_spot has the largest number of samples in the dataset, but its smaller size makes it more difficult to detect. Compared to YOLOv5s, the YOLO-DHGC designed in this paper significantly improved the detection accuracy of Black_spot. This proves that the improvement strategy for small object detection is feasible.

Among the algorithms compared, the YOLO-DHGC algorithm proposed in this paper achieved the highest mAP@0.5 for the five types of backlight board defects. Therefore, compared to mainstream object detection algorithms, YOLO-DHGC effectively detects small defects on the backlight board surface.

**Figure 11:**
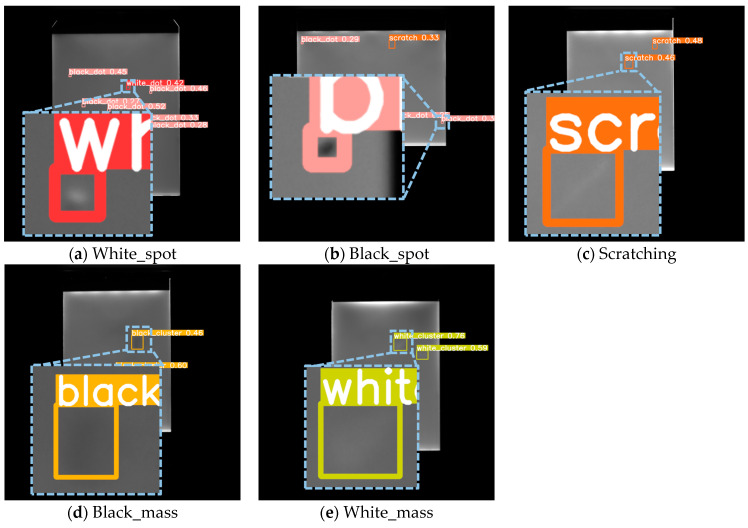
Localized enlargement of detection results of various defects of backlight boards.

Figure 11 shows a partial enlargement of the detection results for various defects in the backlight panel, including White_spot, Black_spot, Scratching, Black_mass, and White_mass. The proposed algorithm effectively detects these defects with minimal misdetections and omissions.

### 4.7. Ablation Experiments

To verify the effectiveness of the proposed two-pronged improvement strategy, ablation experiments were conducted on the PKU-Market-PCB dataset. Starting with the YOLOv5s algorithm, experiments using the DenseHRNet backbone network and the two-stream object detection structure were conducted separately. The results are shown in Table 13 and Table 14.

- Utilization of the DenseHRNet Backbone

Algorithm II is obtained after replacing the modified CSP v5 backbone network of YOLOv5s with the DenseHRNet backbone network. Compared with YOLOv5s, the mAP@0.5, AP_S_, AP_M_, and AP_L_ of algorithm Ⅱ increased by 1.1%, 2.1%, 0.8%, and 1.1%, respectively, the number of model parameters increased by 23.3 M, and the detection speed decreased by 13.8 frames per second. DenseHRNet uses the dense connection mechanism to interact with the multi-resolution feature map, retaining richer small object details and features. This effectively improves the small object detection accuracy. But, the introduction of dense links and the retention of high-resolution feature maps deepen the depth of the network, increasing the number of model parameters and decreasing the detection speed.

- Two-stream Object Detection Structure Based on the Edge-Gated Branch

Algorithm III is obtained by introducing edge-gated streams in parallel with the YOLOv5 backbone network. The output of the edge-gated stream is fused with the backbone network output and fed into the neck network. Compared to YOLOv5s, the mAP@0.5, AP_S_, AP_M_, and AP_L_ of algorithm Ⅲ increased by 1.5%, 2.4%, 0.5%, and 0.5%, respectively; the number of model parameters increased by 88.2M, and the detection speed decreased by 20.5 frames per second. The gated convolutional layer used in the edge-gated branch ensures that the edge-gated stream only processes boundary-related information, filtering out redundant information. This improves localization and detection of small objects. However, the edge-gated stream employs multiple gated convolutional layers with residual learning structure, which increases the number of network parameters and leads to a decrease in detection efficiency.

It is worth noting that, to clearly demonstrate the effect of solely introducing the two-stream structure, we did not use the DenseHRNet backbone in Algorithm III. This approach allows us to more clearly observe the enhancement in detection performance attributed to the two-stream structure itself.

- Using Both the DenseHRNet Backbone and the Edge-Gated Two-stream Detection Structure

Algorithm IV is obtained by combining the DenseHRNet backbone with the edge-gated two-stream detection structure. Experiments show that, compared to YOLOv5s, algorithm IV improves mAP@0.5, AP_S_, AP_M_, and AP_L_ by 1.7%, 2.6%, 0.9%, and 0.6%, respectively. This indicates that the combination of the two enhancement modules further boosts the model’s performance in small object detection. However, the total number of model parameters increased by 111.5M, and the detection speed decreased by 34.3 frames per second. Despite this, the combination not only improves detection accuracy but also significantly enhances overall performance by retaining more details and using boundary information to aid in precise localization of small objects.

## 5. Conclusions

Due to the subtle features of small objects and the complex, variable conditions of detection environments, mainstream deep learning object detection algorithms still face limitations in accurately identifying and localizing tiny targets. To address these issues, this paper proposes a small object detection algorithm based on the YOLO-DHGC framework, aimed at overcoming insufficient information reception and low recognition accuracy. The algorithm uses DenseHRNet as the backbone network and introduces a two-stream structure based on edge-gated mechanisms to enhance detection performance. Specifically, the DenseHRNet backbone combines dense connections with high-resolution feature map branches, strengthening feature reuse and preserving rich details of small objects. Additionally, the two-stream structure based on edge-gated mechanisms integrates gated convolutional layers with image gradient information, effectively capturing clear boundary details of small objects to aid precise localization.

Experiments were conducted on multiple datasets, including PKU-Market-PCB, NEU-DET, TinyPerson, and a self-built backlight panel micro-defect dataset. The proposed YOLO-DHGC algorithm achieved mAP@0.5 scores of 96.3%, 81.6%, 53.98%, and 93.4% on these datasets, improving by 2.0%, 4.7%, 1.53%, and 10.2% over YOLOv5s, respectively. The results demonstrate the effectiveness, robustness, and generalization capabilities of the YOLO-DHGC algorithm in small object detection. Although the proposed YOLO-DHGC algorithm improves the accuracy of small object detection, it also significantly increases the number of model parameters, leading to a decrease in frames per second (FPS). However, in applications that require high-precision detection and have less stringent real-time requirements, this trade-off between performance and resource consumption may be acceptable.

Future research will focus on improving large object detection accuracy and exploring lightweight model strategies to balance accuracy and speed. We will optimize feature extraction to better capture global information and reduce errors. Additionally, we will develop efficient neural networks to lower complexity and parameters, enhancing real-time processing and deployment flexibility without sacrificing performance.

## Figures and Tables

**Table 1:** PKU-Market-PCB dataset information.

Number of Labels/Images	Training Set		Validation Set	Test Set	Totals	
	Original	Enhancement			Original	Enhancement
Missing Hole	340	1987	53	56	449	2327
Mouse Bite	314	1804	49	48	411	2118
Open Circuit	358	2058	50	45	453	2416
Short	340	1998	40	53	433	2338
Spur	385	2258	47	41	473	2643
Spurious Copper	351	2078	51	41	443	2429
Total Number of Images	555	3330	69	69	693	3885

**Table 2:** NEU-DET dataset information.

Number of Labels/Images	Training Set	Validation Set	Test Set	Totals
Crazing, Cr	532	74	83	689
Inclusion, In	732	80	69	881
Patches, Pa	784	116	112	1012
Pitted Surface, Ps	327	47	58	432
Rolled-in Scale, Rs	505	68	55	628
Scratches, Sc	408	65	75	548
Total Number of Images	1440	180	180	1800

**Table 3:** TinyPerson dataset information.

Number of Images or Labels	Training Set	Validation Set	Totals
Number of Images	794	816	1610
Number of Labels	42,197	30,454	72,651

**Table 4:** Information on the backlight panel surface micro-defect data set.

Number of Defects/Images	Training Set		Validation Set	Test Set	Totals	
	Original	Enhancement			Original	Enhancement
White_spot	107	1562	28	24	159	1614
Black_spot	541	7259	42	44	627	7345
Scratching	89	1280	20	19	128	1319
Black_mass	45	628	15	13	73	656
White_mass	84	1202	18	19	121	1239
Total Number of Images	241	3374	31	31	303	3436

**Table 5:** Comparison of detection accuracy of two datasets in different algorithms.

Detection Algorithms	Backbone Network	PCB				NEU-DET			
		mAP@0.5 (%)	AP_S_ (%)	AP_M_ (%)	AP_L_ (%)	mAP@0.5 (%)	AP_S_ (%)	AP_M_ (%)	AP_L_ (%)
YOLOv5s	Modified CSP v5	94.3	45.9	55.1	56.5	76.9	42.7	50.7	51.1
YOLOv8s	CSPDarknet53	93.2	40.4	38.5	51.6	74.7	38.3	33.3	42.1

**Table 6:** Comparison of detection accuracy of different algorithms on the PKU-Market-PCB dataset.

Detection Algorithms	Backbone Network	mAP@0.5 (%)	mAP@0.75 (%)	mAP@0.5:0.95 (%)	AP_S_ (%)	AP_M_ (%)	AP_L_ (%)
Faster R-CNN	ResNet-101	90.5	41.0	50.1	42.7	50.7	51.1
Cascade R-CNN	ResNet-101	91.8	42.6	50.5	44.9	53.1	53.8
SSD512	ResNet-101	780.5	36.5	44.3	37.6	42.8	46.9
RetinaNet	ResNet-101	84.1	40.2	45.9	38.9	47.6	47.3
YOLOv4	CSPDarknet-53	92.7	44.7	51.8	44.8	53	53.1
YOLOv5s	Modified CSP v5	94.3	47.8	52.6	45.9	55.1	56.5
YOLOX-s	Modified CSP v5	92.9	45.7	51.5	45.1	53.4	52.9
YOLOv7s	RepConvN	95.8	48.2	53.3	46.6	55.2	56.3
DETR	ResNet-50	91.2	44.4	49.2	23.3	46.7	46.5
YOLO-DHGC (ours)	DHRNet-W32	**96.3**	**48.5**	**54.0**	**48.5**	**55.4**	**56.9**

**Table 7:** Detection accuracy of each algorithm for six different defects on the PKU-Market-PCB dataset.

Detection Algorithms	Backbone Network	mAP@0.5(%)					
		Missing Hole	Mouse Bite	Open Circuit	Short	Spur	Spurious Copper
Faster R-CNN	ResNet-101	93.9	91.8	88.3	92.6	83.7	92.6
Cascade R-CNN	ResNet-101	94.6	93.7	90.1	94.2	86.7	91.8
SSD512	ResNet-101	91.6	84.6	78.3	85.5	76.3	80.5
RetinaNet	ResNet-101	87.8	85.1	80.6	85.5	79.5	84.4
YOLOv4	CSPDarknet-53	95.3	93.2	92.3	94.1	85.4	93.8
YOLOv5s	Modified CSP v5	96.4	94.9	92.1	95.9	90.7	95.5
YOLOX-s	Modified CSP v5	94.1	93.6	90.4	93.8	88.6	94.1
YOLOv7s	RepConvN	99.1	96.7	94.9	**98.6**	91.5	94.6
DETR	ResNet-50	92.7	91.7	89.9	92.8	86.7	93.1
YOLO-DHGC (ours)	DHRNet-W32	**99.3**	**97.4**	**95.9**	97.2	**91.8**	**96.1**

**Table 8:** Comparison of detection accuracy of different algorithms on NEU-DET dataset.

Detection Algorithms	Backbone Network	mAP@0.5 (%)	mAP@0.75 (%)	mAP@0.5:0.95 (%)	AP_S_ (%)	AP_M_ (%)	AP_L_ (%)
Faster R-CNN	ResNet-101	72.9	40.8	43.1	38.1	40.4	49.1
Cascade R-CNN	ResNet-101	76.3	39.7	43.1	40.8	42.4	50.6
SSD512	ResNet-101	71	34.9	37.4	31.4	32.3	43.5
RetinaNet	ResNet-101	72.5	38.8	40.8	35.5	37.7	44.2
YOLOv4	CSPDarknet-53	75.1	41	45.1	41.4	43.6	50.6
YOLOv5s	Modified CSP v5	76.9	46.2	47.2	41.5	44.6	51.2
YOLOX-s	Modified CSP v5	76.4	42.2	47.5	42.7	42.5	51.5
YOLOv7s	RepConvN	80.2	47.1	49.3	43.1	45.3	**51.8**
YOLO-DHGC (ours)	DHRNet-W32	**81.6**	**47.8**	**50.2**	**44.3**	**45.4**	51.2

**Table 9:** Detection accuracy of each algorithm for six different defects on NEU-DET dataset.

Detection Algorithms	Backbone Network	mAP@0.5 (%)					
		Cr	In	Pa	Ps	Rs	Sc
Faster R-CNN	ResNet-101	40.9	77.4	85.9	82.4	66.5	84.2
Cascade R-CNN	ResNet-101	44.8	78.8	88.8	85.4	70.9	87.3
SSD512	ResNet-101	35.6	78.2	85.6	79.6	60.7	83.2
RetinaNet	ResNet-101	39.8	75.3	84.7	80.6	68.2	84.5
YOLOv4	CSPDarknet-53	41.8	77.5	86.6	85.1	70.4	85.3
YOLOv5s	Modified CSP v5	48.3	82.8	89.4	85.9	75.2	83.9
YOLOX-s	Modified CSP v5	44.4	83.1	87.2	79.5	**80.0**	86.3
YOLOv7s	RepConvN	50.2	**86.5**	90.4	85.4	79.2	84.5
YOLO-DHGC (ours)	DHRNet-W32	**51.1**	85.8	**91.5**	**87.9**	78.9	**87.4**

**Table 10:** Comparison of detection accuracy rates of different algorithms in TinyPerson dataset.

Detection Algorithms	Backbone Network	$AP_{0.5}^{\mathrm{tiny}}$ (%)	$AP_{0.5}^{\mathrm{tiny}1}$ (%)	$AP_{0.5}^{\mathrm{tiny}2}$ (%)	$AP_{0.5}^{\mathrm{tiny}3}$ (%)	$AP_{0.5}^{\mathrm{small}}$ (%)	$AP_{0.25}^{\mathrm{tiny}}$ (%)	$AP_{0.75}^{\mathrm{tiny}}$ (%)
Faster R-CNN	ResNet-101	48.01	29.45	50.25	57.69	63.29	69.32	6.03
Cascade R-CNN	ResNet-101	51.12	31.78	53.21	60.38	65.12	72.01	6.42
SSD512	ResNet-101	34.12	13.48	35.29	48.83	57.36	61.67	2.67
RetinaNet	ResNet-101	45.52	26.63	51.01	55.78	57.38	68.24	4.16
YOLOv4	CSPDarknet-53	50.33	32.15	52.67	58.36	66.92	71.24	6.26
YOLOv5s	Modified CSP v5	52.45	35.57	54.23	61.02	65.32	73.45	6.63
YOLOX-s	Modified CSP v5	53.57	37.31	57.37	63.95	67.31	75.82	7.30
YOLOv7s	RepConvN	52.67	35.69	55.67	62.13	66.35	74.72	6.91
**YOLO-DHGC (ours)**	DHRNet-W32	**53.98**	**38.43**	**58.91**	**64.23**	**67.92**	**76.15**	**7.39**

**Table 11:** Comparison of detection accuracy of different algorithms on self-built backlight panel micro-defect dataset.

Detection Algorithms	Backbone Network	mAP@0.5 (%)	mAP@0.5:0.95 (%)	AP_S_ (%)	AP_M_ (%)	AP_L_ (%)
Faster R-CNN	ResNet-101	78.1	38.6	33.2	35.8	48.3
Cascade R-CNN	ResNet-101	79.9	41.2	35.8	39.1	54.3
SSD512	ResNet-101	71.3	34.7	29.3	29.4	46.9
RetinaNet	ResNet-101	76.2	36.2	30.8	33.2	48.6
YOLOv4	CSPDarknet-53	78.3	39.8	34.4	37.5	50
YOLOv5s	Modified CSP v5	83.2	43.2	38.2	42.6	56.7
YOLOX-s	Modified CSP v5	85.5	44.8	39.4	46.2	57.2
YOLOv7s	RepConvN	89.3	49.9	44.1	50.9	63.2
**YOLO-DHGC (ours)**	DHRNet-W32	**93.4**	**52.8**	**47.4**	**53.7**	**64.8**

**Table 12:** Accuracy of each algorithm for five types of defects on the self-built backlight panel micro-defect dataset.

Detection Algorithms	Backbone Network	mAP@0.5 (%)				
		White_spot	Black_spot	Scratching	Black_mass	White_mass
Faster R-CNN	ResNet-101	77.5	67.3	82.5	76.3	83.4
Cascade R-CNN	ResNet-101	80.4	63.3	78.4	74.8	87.8
SSD512	ResNet-101	73.5	51.3	69.8	69.5	85.4
RetinaNet	ResNet-101	81.4	55.1	73.8	72.5	87.3
YOLOv4	CSPDarknet-53	79.3	60.2	80.1	72.3	90.4
YOLOv5s	Modified CSP v5	85.2	64.1	84.6	75.9	94.4
YOLOX-s	Modified CSP v5	85.8	62.5	87.2	78.5	97.9
YOLOv7s	RepConvN	86.9	76.7	93.5	96.1	98.6
**YOLO-DHGC (ours)**	**DHRNet-W32**	**90.4**	**79.3**	**96.4**	**98.2**	**98.9**

**Table 13:** Accuracy ablation experiments with five improved strategies. The check symbols (✔) in the table indicate the presence of the corresponding improvement strategies in the respective algorithms.

Algorithm Number	Improvement Strategies		mAP@0.5 (%)	AP_S_ (%)	AP_M_ (%)	AP_L_ (%)
	DenseHRNet	Two-Stream				
Ⅰ	YOLOV5s (baseline)		94.3	45.9	54.5	56.7
Ⅱ	✔		95.4	48	55.3	57.8
Ⅲ		✔	95.8	48.3	55.0	57.2
Ⅳ	✔	✔	96.3	48.5	55.4	56.3

**Table 14:** Model complexity ablation experiments with five improvement strategies. The check symbols (✔) in the table indicate the presence of the corresponding improvement strategies in the respective algorithms.

Algorithm Number	Improvement Strategies		mAP@0.5 (%)	Params (M)	FPS (Fps)
	DenseHRNet	Two-Stream			
I	YOLOV5s (baseline)		94.3	13.5	49.1
II	✔		95.4	36.8	35.3
III		✔	95.8	101.7	28.6
IV	✔	✔	96.3	133.6	17.7

## Data Availability

The data presented in this study are available on request from the corresponding authors.
